# 高效液相色谱-串联质谱法测定化妆品中3种甲基咪唑类化合物

**DOI:** 10.3724/SP.J.1123.2023.11011

**Published:** 2024-11-08

**Authors:** Cuiling CAI, Ju LI, Jing XI, Yingye HOU, Guangfeng ZENG, Jianjun XIE, Shuyue WU, Lu WANG

**Affiliations:** 广州海关技术中心, 国家级进出口食品质量安全风险验证评价实验室(化妆品), 广东 广州 510623; Guangzhou Customs Technical Center, National Import and Export Food Quality Safety Risk Verification and Evaluation Laboratory (Cosmetics), Guangzhou 510623, China

**Keywords:** 高效液相色谱-串联质谱, 1-甲基咪唑, 2-甲基咪唑, 4-甲基咪唑, 化妆品, high performance liquid chromatography-tandem mass spectrometry (HPLC-MS/MS), 1-methylimidazole (1-MEI), 2-methylimidazole (2-MEI), 4-methylimidazole (4-MEI), cosmetics

## Abstract

建立了一种同时测定化妆品中1-甲基咪唑(1-MEI)、2-甲基咪唑(2-MEI)和4-甲基咪唑(4-MEI)的高效液相色谱-串联质谱(HPLC-MS/MS)方法。样品经乙腈提取、混合型阳离子交换(MCX)固相萃取柱净化、氮吹及复溶后,使用XBridge^®^ shield RP_18_色谱柱(150 mm×4.6 mm, 3.5 μm)进行分离;以含0.1%甲酸的20 mmol/L甲酸铵水溶液-乙腈(98∶2, v/v)作为流动相进行等度洗脱;质谱分析采用电喷雾电离(ESI)源和多反应监测(MRM)模式,外标法定量。结果表明,1-MEI在5~200 μg/L范围内线性关系良好(相关系数(*r*^2^)≥0.9994), 2-MEI和4-MEI在2~100 μg/L范围内线性关系良好(*r*^2^≥0.9984)。3种甲基咪唑类化合物的检出限(LOD)为10~30 μg/kg,定量限(LOQ)为25~100 μg/kg。在低、中、高3个加标水平下,不同空白基质中3种甲基咪唑类化合物的回收率为80.9%~107.9%,相对标准偏差(RSD, *n*=6)为1.2%~12.8%。将该方法应用于48个化妆品样品的检测,结果表明,有9个样品检出4-MEI,检出含量为26~1000 μg/kg; 2个样品检出2-MEI,检出含量分别为240 μg/kg和267 μg/kg;而48个样品中均未检出1-MEI。该方法简单、快速,灵敏度高,为化妆品中3种甲基咪唑类化合物的风险评估和筛查监测提供了技术支撑。

## Abstract (en)

Methylimidazole compounds are byproducts formed during the caramel-coloring process and are used in various cosmetics. In addition metronidazole is an antibacterial and anti-inflammatory drug commonly used in modern medicine and is used in cosmetics to treat acne in the short-term. The illegal addition of metronidazole during cosmetics production can result in residual 2-methylimidazole (2-MEI), which, along with 4-methylimidazole (4-MEI), is a class 2B carcinogen. Therefore, establishing efficient, accurate, and sensitive analytical techniques for analyzing methylimidazole compounds in cosmetics is an urgent objective. In this study, a high performance liquid chromatography-tandem mass spectrometry (HPLC-MS/MS) method for simultaneously determining 1-methylimidazole (1-MEI), 2-MEI, and 4-MEI in cosmetics was developed. Cosmetics samples were extracted via ultrasonication in acetonitrile and purified using a mixed cation-exchange (MCX) solid-phase extraction (SPE) column, with subsequent drying under a stream of nitrogen and redissolution in acetonitrile. The resulting solution was then filtered through a 0.22 μm organic filter membrane for further testing. The analytes were separated using an XBridge^®^ shield RP_18_ chromatographic column (150 mm×4.6 mm, 3.5 μm) and isocratically eluted with 20 mmol/L ammonium formate solution (containing 0.1% formic acid)-acetonitrile (98∶2, v/v). The target compounds were ionized by electrospray ionization (ESI) source, analyzed in multi-reaction monitoring (MRM) mode, and quantified using the external standard method, with the peak area of the quantitative ion and the mass concentration of the compound taken as the longitudinal and transverse coordinates, respectively. Matrix-matching working curves were also constructed. 1-MEI exhibited good linear relationships in the range of 5-200 μg/L, with correlation coefficients (*r*^2^)≥0.9994, while 2-MEI and 4-MEI showed good linearities in the range of 2-100 μg/L with *r*^2^≥0.9984. The three methylimidazole compounds exhibited limits of detection (LODs) and quantification (LOQs) of 10-30 μg/kg and 25-100 μg/kg, respectively. Under three spiked levels (LOQ, 2LOQ, 10LOQ), the recoveries of three methylimidazole compounds were 80.9%-107.9%, with relative standard deviations (RSDs, *n*=6) of 1.2%-12.8%. The practicability of the method was examined using 48 cosmetic samples; 4-MEI was detected in nine samples at contents of 26-1000 μg/kg, while two samples contained 240 and 267 μg/kg of 2-MEI, respectively. 1-MEI was not detected in any of the 48 samples tested. The developed method is simple, fast, and highly sensitive, and provides methodological support for assessing risks and monitoring the three methylimidazole compounds in cosmetics through screening.

甲基咪唑类化合物是焦糖色素生产过程中的副产物^[1]^,而焦糖色素被允许在各类化妆品中使用。据文献[2,3]报道,4-甲基咪唑(4-methylimidazole, 4-MEI)可抑制细胞色素P450同工酶的活性,诱发动物惊厥,存在致癌风险。甲硝唑是一类抗菌消炎药,其价格低廉,在临床上主要用于疥疮、足癣等皮肤病的治疗;若在祛痘类化妆品的生产过程中非法添加甲硝唑,在短期内可产生明显的祛痘效果。我国《化妆品安全技术规范》(2015版)中明确规定,甲硝唑为化妆品禁用物质,不得作为化妆品的生产原料和组分添加到化妆品中^[4]^。2-甲基咪唑(2-methylimidazole, 2-MEI)是甲硝唑的合成中间体,若在化妆品中违法添加甲硝唑会导致2-MEI的残留,反复使用此类化妆品会诱发皮肤菌群失调,引起致敏性反应(如接触性皮炎、抗生素过敏等),长期使用还会导致严重的内脏损伤。2017年,世界卫生组织国际癌症研究机构公布了致癌物清单,其中2-MEI和4-MEI均被定义为2B类致癌物^[5]^。1-甲基咪唑(1-methylimidazole, 1-MEI)与2-MEI、4-MEI互为同分异构体,具有相似的物理、化学性质,其危害性同样也应受到关注。

目前,甲基咪唑类化合物的检测标准和方法主要集中在食品领域,而化妆品领域则较少。甲基咪唑类化合物的检测方法主要包括气相色谱法^[6]^、气相色谱-质谱法^[7⇓-9]^、高效液相色谱法^[10]^和液相色谱-串联质谱法^[11⇓⇓⇓-15]^。闵宇航等^[1]^基于超高效液相色谱-串联质谱建立了酱卤肉制品中1-MEI、2-MEI和4-MEI同时测定的方法;王彬潆等^[12]^基于超高效液相色谱-串联质谱建立了客家娘酒中2-MEI、4-MEI和5-羟甲基糠醛的分析方法。由于化妆品的基质种类较多、添加组分复杂,因此不可简单套用食品中甲基咪唑类化合物的提取、净化方法。

本研究基于高效液相色谱-串联质谱(HPLC-MS/MS)建立了1-MEI、2-MEI和4-MEI的快速、准确检测方法,可同时检测多种化妆品基质中的3种甲基咪唑类化合物,为化妆品的风险评估、进出口贸易监管及安全管理等提供技术支撑,保障消费者的化妆品使用安全。

## 1 实验部分

### 1.1 仪器、试剂与材料

API 4000Q Trap HPLC-MS/MS(美国AB公司); Milli-Q超纯水系统(美国Millipore公司); NMSG-12多管涡旋混合器(泰州诺米医疗科技有限公司); BiofugePrimoR高速离心机(美国赛默飞公司); PS-80超声波清洗器(东莞市洁康超声波设备有限公司); TurboVap多功能全自动样品浓缩仪(Biotage公司)。

甲醇、乙腈、无水乙醇、甲酸(均为色谱纯),购自美国Thermo Fisher公司; 1-MEI、2-MEI、4-MEI标准品(纯度≥98%),购自上海安谱璀世标准技术服务有限公司;甲酸铵(色谱纯)、混合型阳离子交换(MCX)固相萃取柱(3 mL/60 mg)、混合型弱阳离子交换(WCX)固相萃取柱(3 mL/60 mg)、氨丙基(NH_2_)萃取柱(6 mL/500 mg)、增强型脂质去除(EMR-Lipid)固相萃取柱(3 mL/300 mg),购自上海安谱实验科技股份有限公司;28%氨水购自广州化学试剂厂。

化妆品样品均为实验室送检样品,包括水基类(爽肤水)、油基类(精油)、膏霜乳类(面霜、精华乳、洗发水、染发剂、粉底液)、粉基类(散粉、眼影)等。

### 1.2 溶液的配制

分别准确称取10 mg(精确至0.01 mg)1-MEI、2-MEI和4-MEI标准品,用乙腈溶解,配制成质量浓度为1000 mg/L的混合标准储备液,于4 ℃下避光保存;用乙腈对混合标准储备液进行稀释,配制成质量浓度为1 mg/L的混合标准溶液,用于后续的加标回收试验。临用前,用空白基质提取液对混合标准储备液进行逐级稀释,配制成系列质量浓度(2、5、10、20、50、100、200 μg/L)的基质匹配混合标准溶液。

### 1.3 样品前处理

#### 1.3.1 提取

水基类、油基类、膏霜乳类样品的提取:准确称取0.2 g(精确至0.001 g)样品于50 mL离心管中,加入10 mL乙腈,涡旋振荡2 min,使样品与提取溶剂充分混匀;超声提取15 min,于10000 r/min下离心5 min,将全部上清液转移至玻璃管中,氮吹至溶液体积约为2 mL,待净化。

粉基类样品的提取:准确称取0.2 g(精确至0.001 g)样品于50 mL离心管中,加入1 mL饱和氯化钠溶液,使样品均匀分散,再加入10 mL乙腈,随后的提取步骤与水基类等完全一致。

#### 1.3.2 净化

依次使用1 mL甲醇和1 mL超纯水对MCX固相萃取柱进行活化,取1.3.1节中的待净化液,全部过柱,并控制流速为1 mL/min;依次用1 mL超纯水和1 mL甲醇对MCX固相萃取柱进行淋洗,再用3 mL 28%氨水-甲醇(5∶95, v/v)进行洗脱,收集洗脱液于玻璃管中,于45 ℃水浴中氮吹至干,再用乙腈定容至1 mL,涡旋混匀,过0.22 μm有机滤膜,滤液供HPLC-MS/MS测定。

### 1.4 分析条件

#### 1.4.1 色谱条件

色谱柱:XBridge^®^ shield RP_18_柱(150 mm×4.6 mm, 3.5 μm);流动相为含0.1%甲酸的20 mmol/L甲酸铵水溶液-乙腈(98∶2, v/v),等度洗脱5 min;流速:0.6 mL/min;进样量:20 μL;柱温:45 ℃。

#### 1.4.2 质谱条件

离子源:电喷雾电离(ESI)源;扫描方式:正离子扫描;监测模式:多反应监测(MRM);电喷雾电压:5500 V;离子源温度:550 ℃;气帘气压力:103425 Pa;雾化气压力:344750 Pa;加热辅助气压力:344750 Pa。3种甲基咪唑类化合物的保留时间和质谱参数见表1。

**表1:** 3种甲基咪唑类化合物的保留时间和质谱参数

Compound	Retention time/min	Precursor ion (*m/z*)	Product ion (*m/z*)	DP/V	CE/eV
1-Methylimidazole (1-MEI)	2.71	83.2	56.1^*^	55	26.9
			42.2	55	39.9
2-Methylimidazole (2-MEI)	2.85	83.2	42.2^*^	55	39.9
			56.1	55	26.9
4-Methylimidazole (4-MEI)	3.05	83.2	56.1^*^	55	26.9
			42.2	55	39.9

*Quantitative ion; DP: declustering potential; CE: collision energy.

## 2 结果与讨论

### 2.1 色谱条件的优化

#### 2.1.1 色谱柱的选择

本实验比较了Atlantis HILIC Silica(100 mm×3.0 mm, 3.0 μm)、ZORBAX SB-Aq(100 mm×4.6 mm, 3.5 μm)、XBridge^®^ shield RP_18_(150 mm×4.6 mm, 3.5 μm)3种色谱柱对1-MEI、2-MEI和4-MEI的分离效果。Atlantis HILIC Silica柱属于亲水作用色谱柱,其在亲水作用下对强极性化合物有良好的保留。在甲基咪唑类化合物检测的文献报道中,亲水作用色谱柱最为常用^[1,12⇓-14]^。本研究发现,Atlantis HILIC Silica柱对3种甲基咪唑类化合物的分离度虽然很高,但其出峰稳定性及重复性却较差,容易出现色谱峰分叉的现象,尤其是当使用含有甲酸铵的缓冲液作为流动相时,上述现象更加明显;分析其原因,可能是亲水作用色谱柱在维持色谱峰稳定性和重复性方面需要较长的平衡时间。ZORBAX SB-Aq柱具有亲水表面,是一种HPLC烷基反相键合相,对亲水性化合物具有很好的保留,但本实验发现,待测物经ZORBAX SB-Aq柱分离后会产生色谱峰形展宽和拖尾的现象。XBridge^®^shield RP_18_柱含极性内嵌C_18_链,能够有效改善色谱峰形,实验结果表明,3种甲基咪唑类化合物在XBridge^®^ shield RP_18_柱上均可实现良好的基线分离,且色谱峰峰形良好,稳定性好。因此,本方法最终选择XBridge^®^ shield RP_18_色谱柱。

#### 2.1.2 流动相的优化

实验考察了5种不同流动相(0.1%甲酸水溶液-乙腈、含0.1%甲酸的5 mmol/L甲酸铵水溶液-乙腈、含0.1%甲酸的20 mmol/L甲酸铵水溶液-乙腈、5 mmol/L甲酸铵水溶液-乙腈、20 mmol/L甲酸铵水溶液-乙腈)对1-MEI、2-MEI和4-MEI分离效果和响应强度的影响,其中水相与有机相的体积比均为98∶2。结果表明,在甲酸铵水溶液中添加甲酸后,待测物的色谱峰形和分离度均得到了改善和提高;在甲酸添加量相同的高浓度(20 mmol/L)甲酸铵水溶液中,待测物的色谱峰形和分离度更佳,而没有添加甲酸铵的流动相对3种甲基咪唑类化合物的分离效果较差。分析其原因,甲酸铵的加入可以使待测物在质谱中的离子化程度变高,质谱响应更强;同时,作为一种缓冲盐,甲酸铵有利于提高分离度,改善色谱峰形^[16]^。因此,选择含0.1%甲酸的20 mmol/L甲酸铵水溶液-乙腈(98∶2, v/v)作为流动相。

### 2.2 前处理条件的优化

#### 2.2.1 提取溶剂的选择

3种甲基咪唑类化合物均易溶于水和有机溶剂,本实验考察了超纯水、无水乙醇、甲醇和乙腈4种提取溶剂对1-MEI、2-MEI和4-MEI提取效果的影响。在水基类、油基类、霜膏乳类、粉基类4类化妆品基质中分别选择爽肤水、精油、面霜和散粉作为代表,进行空白基质加标(50 μg/L)试验。实验结果表明,与超纯水和乙腈相比,采用甲醇和无水乙醇进行提取时,爽肤水中1-MEI和4-MEI的回收率偏低;与其他3种溶剂相比,采用无水乙醇进行提取时,精油中4-MEI的回收率最低(<80%);与其他3种溶剂相比,采用纯水进行提取时,面霜中1-MEI的回收率最低,仅为76.3%。此外,若将纯水作为提取溶剂,后期净化时难以吹干;同时实验过程中还发现,用甲醇和无水乙醇提取面霜时会出现絮状物,难以自然过柱且过柱时间较长。综合考虑上述因素,选择乙腈作为3种甲基咪唑类化合物的提取溶剂。

对于散粉类化妆品,单独采用乙腈进行提取时,散粉中3种甲基咪唑类化合物的回收率仅为11.5%~45.5%,说明单纯使用有机溶剂无法充分分散散粉类化妆品,不能达到良好的提取效果。针对该类基质,本实验以饱和氯化钠溶液作为分散溶剂,之后再用乙腈进行提取。结果表明,散粉中3种甲基咪唑类化合物的回收率为81.2%~86.5%,能够满足实际测定要求。

#### 2.2.2 固相萃取柱的选择

实验比较了不同净化柱(MCX固相萃取柱、WCX固相萃取柱、NH_2_萃取柱和EMR-Lipid固相萃取柱)对3种甲基咪唑类化合物的净化效果。分别在爽肤水、精油和面霜3种空白基质提取液中添加3种甲基咪唑类化合物的混合标准溶液(50 μg/L)进行加标回收试验,并以回收率作为考察指标。由于4种固相萃取柱的性能不同,本实验参照文献[1,13]和固相萃取柱使用说明书,采用了不同的净化操作。结果如图1所示,当使用MCX固相萃取柱进行净化时,爽肤水、精油和面霜基质中3种甲基咪唑类化合物的回收率为80.0%~95.3%;当使用WCX固相萃取柱和NH_2_萃取柱进行净化时,爽肤水基质中3种甲基咪唑类化合物的回收率为71.4%~98.0%,但WCX固相萃取柱对精油和面霜基质中3种甲基咪唑类化合物的吸附作用极强,回收率仅为0~3.93%; NH_2_萃取柱对精油和面霜基质中3种甲基咪唑类化合物的提取效果影响也较大,回收率为21.1%~326.0%; EMR-Lipid固相萃取柱的净化过程虽然简单,但其对3种待测物的吸附作用较强,所得到的回收率仅为0~58.2%,无法满足测定需求。因此,最终选择MCX固相萃取柱作为3种甲基咪唑类化合物的净化柱。

**图1:**
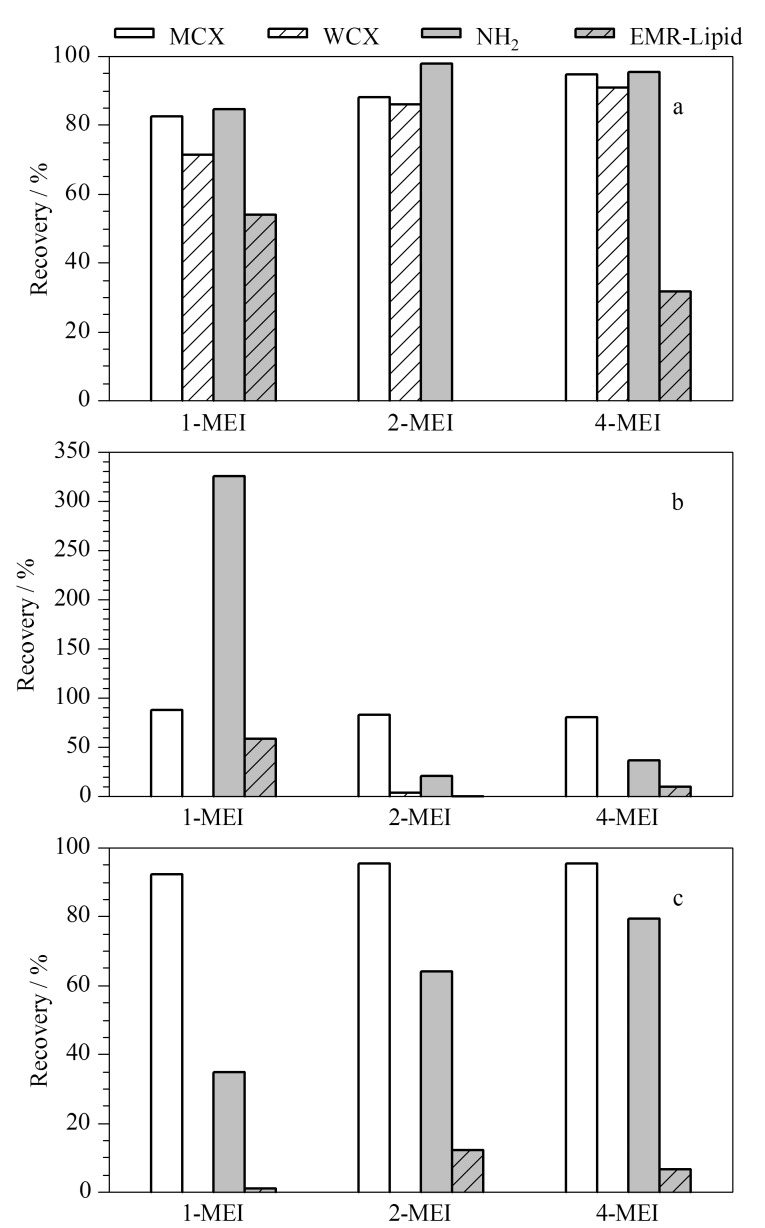
不同净化柱对(a)爽肤水、(b)精油、(c)面霜基质中3种甲基咪唑类化合物回收率的影响

#### 2.2.3 洗脱溶剂体积的优化

为减少有机试剂的消耗并提升待测物的提取效率,实验考察了不同体积(1、2、3、4、5 mL)的28%氨水-甲醇(5∶95, v/v)对3种甲基咪唑类化合物洗脱效率的影响。实验结果表明,当洗脱溶剂的体积为3 mL时,1-MEI、2-MEI和4-MEI的回收率达到最大,而继续增大洗脱溶剂的体积,3种待测物的回收率无明显变化,因此确定洗脱溶剂的体积为3 mL。在最佳实验条件下,3种甲基咪唑类化合物的总离子流色谱图见图2。

**图2:**
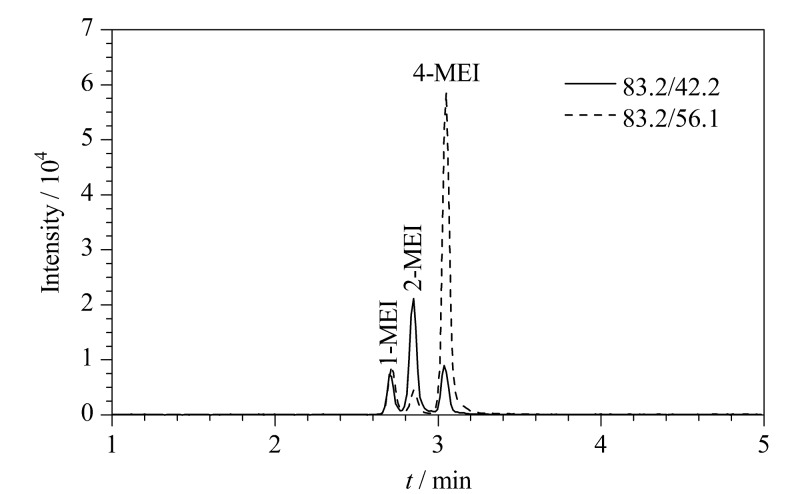
3种甲基咪唑类化合物在最佳实验条件下的总离子流色谱图

### 2.3 基质效应

基质效应(matrix effect, ME)是指样品基质对待测物分析结果的影响,其计算公式为ME=(基质匹配混合标准曲线斜率/溶剂标准曲线斜率-1)×100%。若|ME|<20%,表示存在弱基质效应;20%≤|ME|<50%,表示存在中等基质效应;|ME|≥50%,表述存在强基质效应^[17]^。本实验以爽肤水、精油、面霜为代表,制备了相应的空白基质提取液,考察3种样品基质的基质效应。结果表明,在爽肤水基质中,1-MEI和4-MEI表现出弱基质效应,2-MEI表现出中等基质效应;在精油基质中,1-MEI和2-MEI表现出弱基质效应,4-MEI表现出强基质效应;在面霜基质中,1-MEI和2-MEI表现出弱基质效应,4-MEI表现出中等基质效应。因此,为尽量减少基质效应的干扰,本方法采用基质匹配混合标准溶液来进行后续的定量实验。

### 2.4 线性范围、检出限和定量限

分别用爽肤水、精油、面霜和散粉4种空白基质提取液对3种甲基咪唑类化合物的混合标准储备液进行稀释,配制成系列质量浓度的基质匹配混合标准溶液,按优化后的方法进样分析。以定量离子对的峰面积为纵坐标(*Y*),对应的质量浓度为横坐标(*X*, μg/L),分别绘制基质匹配工作曲线。实验结果表明,在4种化妆品基质中,1-MEI在5~200 μg/L范围内线性关系良好(相关系数(*r*^2^)≥0.9994), 2-MEI和4-MEI在2~100 μg/L范围内线性关系良好(*r*^2^≥0.9984)。分别以3倍信噪比(*S/N*=3)和*S/N*=10所对应的质量浓度作为检出限(LOD)和定量限(LOQ), 3种甲基咪唑类化合物的LOD为10~30 μg/kg, LOQ为25~100 μg/kg,相关数据详见表2。

**表2:** 3种甲基咪唑类化合物的线性范围、线性方程、相关系数、检出限和定量限

Compound	Linear range/ (μg/L)	Toner matrix			Volatile oil matrix			Cream matrix			Loose powder matrix		LOD/ (μg/kg)	LOQ/ (μg/kg)
		Linear equation	*r*^2^		Linear equation	*r*^2^		Linear equation	*r*^2^		Linear equation	*r*^2^		
1-MEI	5-200	*Y*=254*X*+9	0.9996		*Y*=264*X*+307	0.9994		*Y*=235*X*+1320	0.9996		*Y*=358*X*+3210	0.9998	30	100
2-MEI	2-100	*Y*=1140*X*-284	0.9996		*Y*=1320*X*+1570	0.9994		*Y*=1180*X*+2420	0.9990		*Y*=1310*X*+2200	0.9984	10	25
4-MEI	2-100	*Y*=3210*X*+5620	0.9998		*Y*=3240*X*+9870	0.9991		*Y*=3260*X*+8480	0.9998		*Y*=2370*X*+30000	0.9996	10	25

*Y*: peak area; *X*: mass concentration, μg/L.

### 2.5 回收率和精密度

选取有代表性的空白样品基质(爽肤水、精油、面霜、散粉)来考察所建方法的实用性。分别向4种空白基质提取液中添加低、中、高(LOQ、2LOQ、10LOQ)3个水平的混合标准溶液,进行加标回收试验,每个加标水平平行测定6次,并计算回收率和相对标准偏差(RSD),结果见表3。在4种化妆品基质中,3种甲基咪唑类化合物的回收率为80.9%~107.9%,RSD为1.2%~12.8%,其中10LOQ加标水平下的回收率略低于LOQ、2LOQ加标水平,这可能是因为10LOQ加标水平已接近该方法的检出上限。上述结果表明,本文所建方法能够满足日常检测需求。

**表3:** 3种甲基咪唑类化合物在4种化妆品基质中的回收率和相对标准偏差(*n*=6)

Compound	Spiked level/ (μg/kg)	Toner matrix			Volatile oil matrix			Cream matrix			Loose powder matrix	
		Recovery/%	RSD/%		Recovery/%	RSD/%		Recovery/%	RSD/%		Recovery/%	RSD/%
1-MEI	100	90.8	5.5		84.0	3.3		89.7	3.7		83.0	2.7
	200	91.3	10.1		85.4	5.3		84.4	3.2		83.1	3.6
	1000	82.0	1.2		83.8	2.2		83.7	2.2		80.9	1.9
2-MEI	25	107.9	8.8		87.9	9.4		93.0	5.0		90.2	5.0
	50	104.7	4.2		91.7	5.8		101.7	11.3		84.7	3.8
	250	87.8	6.6		88.6	3.4		85.6	6.0		84.8	2.3
4-MEI	25	94.4	12.2		93.8	12.8		85.5	5.3		85.5	5.6
	50	89.3	2.2		100.8	4.2		101.4	9.6		89.4	7.2
	250	86.4	6.7		89.5	5.7		82.8	2.8		84.9	2.3

### 2.6 实际样品检测

采用所建立的方法对爽肤水、精油、面霜、精华乳、洗发水、染发剂、粉底液、散粉、眼影等48个化妆品样品进行测定。结果发现,所有样品均未检出1-MEI; 9个样品(2款染发剂、7款霜膏乳类)检出4-MEI,检出含量为26~1000 μg/kg; 2个样品(2款染发剂)检出2-MEI,检出含量分别为240 μg/kg和267 μg/kg。实验结果表明,本文所建方法对市场上大部分化妆品都具备一定的检测能力,能够实现化妆品中3种甲基咪唑类化合物的风险监控。

## 3 结论

本研究基于高效液相色谱-串联质谱建立了化妆品中3种甲基咪唑类化合物(1-MEI、2-MEI和4-MEI)的同时测定方法。该方法简单、快速,灵敏度和准确度高,实现了不同基质化妆品中3种甲基咪唑类化合物的同时检测。该方法可为打击化妆品非法添加、保障化妆品质量安全及开展化妆品风险评估等工作提供技术支撑,对加强市场监管、进出口贸易及安全管理具有重要意义。
